# Exploring plausible futures for artificial intelligence in rural healthcare: insights from participatory foresight methods

**DOI:** 10.3389/fdgth.2026.1750172

**Published:** 2026-04-13

**Authors:** Tara Cain, Rachel Curtis, Ben Singh, Ashleigh E. Smith, Jacinta Brinsley, Carol Maher, Aaron Davis

**Affiliations:** 1 Alliance for Research in Exercise Nutrition and Activity (ARENA), UniSA Allied Health and Human Performance, University of South Australia, Adelaide, SA, Australia; 2 UniSA Creative, University of South Australia, Adelaide, SA, Australia

**Keywords:** artificial intelligence (AI), digital health, equitable access, foresight methods, participatory research, rural healthcare, stakeholder engagement

## Abstract

**Background:**

Artificial intelligence (AI) has the potential to transform rural healthcare delivery through automated monitoring, personalised care, and virtual support. Yet the future pathways for AI in rural contexts remain underexplored. Most AI applications are developed in urban-centric environments with limited consideration for infrastructure constraints, workforce realities, and sociocultural dynamics that shape rural healthcare delivery.

**Methods:**

This study examined stakeholder perspectives on the future role of AI in rural healthcare, identifying key priorities, facilitators, and barriers to adoption. Using a participatory research approach incorporating horizon scanning and foresight methods, data were collected during a structured workshop at the South Australian Rural Health Research and Education Conference. Forty participants, including general practitioners, clinicians, medical students, researchers, and healthcare administrators, engaged in four sequential activities: historical events mapping, future event possibilities, experiential future scenarios, and priority setting using the MoSCoW framework. Written responses were systematically transcribed and analysed using reflexive thematic analysis.

**Results:**

Four prominent themes emerged capturing stakeholder priorities and the guardrails they considered essential for future technological integration. These themes related to opportunities from AI and technology deployment for rural and remote equity, people at the centre of care, ethical challenges, and funding and systems issues. Participants acknowledged AI's potential to reduce geographical barriers and improve access to healthcare services, while also raising concerns about data privacy, governance, cultural appropriateness, and the risk of technology exacerbating existing health disparities. Across activities, participants expressed a strong preference for AI that supports rather than replaces human clinicians, and emphasised the importance of maintaining person-centred care, human connection, and local knowledge.

**Discussion:**

This study shows how futures-oriented, participatory methods can surface both the promise and the constraints of AI in rural healthcare. Successful implementation requires co-design with rural communities, equity-driven approaches, transparent governance frameworks, and investment in infrastructure and workforce capacity so that future technology adoption supports, rather than exacerbates, existing health disparities.

## Introduction

Artificial intelligence (AI) is increasingly positioned as a transformative force in healthcare, offering improvements in diagnostic precision, clinical decision-making, and health system operations. Applications such as medical image analysis, personalised treatment planning, and predictive modelling using electronic health records have gained widespread attention (1–3). More recently, AI has been used to analyse data from wearable devices and in home-monitoring systems, generating insights into behaviours like physical activity and medication adherence (4). Within healthcare management, AI-enabled systems have improved clinical accuracy, streamlined decision-making, and enhanced operational efficiency through predictive analytics and automation (4).

Rural healthcare settings present persistent challenges that digital technologies might help address. These include geographic isolation, workforce shortages, and limited access to specialised care. These disparities are pronounced in the Australian context: people living in very remote areas have a mortality rate 1.5 times the national average, and the rate of potentially avoidable deaths is up to 3.4 times higher than in major cities (5). Access to primary care is also constrained, with people in very remote communities averaging just 3.3 GP visits per person annually compared to 6.3 in metropolitan areas (5). Digital health interventions such as telehealth have shown potential to reduce some of these barriers by facilitating virtual consultations and enabling remote monitoring. AI technologies may extend this promise further through context-aware decision support and adaptive care models that tailor recommendations to local contexts and patient needs, particularly in areas such as chronic disease management (6).

Despite growing interest in integrating AI into healthcare systems, the unique requirements of rural healthcare integration remain unexplored. Much of the literature continues to emphasise system-wide efficiency, clinical accuracy, and technological advancement, often at the expense of investigating contextual factors that influence adoption and equity (7, 8). Emerging findings have highlighted that AI applications are predominantly developed in urban-centric environments, with limited consideration for the infrastructure constraints, workforce challenges, and sociocultural dynamics that define rural healthcare delivery (9, 10). In the Australian context, this includes the cultural values and practices of First Nations communities, varying levels of digital literacy, and differing community expectations around trust and relational care (11). Participatory approaches that engage rural stakeholders in shaping AI solutions are rarely prioritised (12), despite growing recognition that these approaches ensure relevance, build trust, and enhance local legitimacy (13). Furthermore, the risks associated with AI implementation may be uniquely amplified in rural settings. For example, in areas with persistent workforce shortages, AI tools may be used to replace rather than supplement in-person care, potentially risking professional and in-person care.

The aim of this study was to collaboratively explore potential future roles of AI in rural healthcare through structured engagement with health care (administrators, general practitioners, clinicians, medical students) and research stakeholders. Specifically, our study sought to: i) explore stakeholder perceptions of significant historical developments in digital health within rural contexts; ii) explore potential future AI applications designed to address rural healthcare challenges; iii) develop experiential scenarios to contextualise these possibilities; and iv) surface stakeholder-driven priorities for AI integration in rural healthcare settings.

## Methods

### Study design, reflexivity statement, and ethics

This study employed a participatory research approach incorporating horizon scanning and strategic foresight methods (14) to collaboratively explore potential future roles AI may have in rural healthcare. These approaches are particularly suited to exploring uncertain, values-laden topics in settings where future trajectories are contested and stakeholder engagement is essential for legitimacy (14). Given the exploratory and futures focussed nature of the study, a constructivist paradigm based on the idea that reality and futures are not objective and fixed but rather constructed by individuals through their own diverse perspectives and shaped by experiences was adopted.

The study was funded by a Medical Research Future Fund Maternal Health and Healthy Lifestyles grant (GNT2031344) which aims to co-design and evaluate an AI-powered digital health coaching program that delivers personalised lifestyle support, with a focus on diet, physical activity, and weight management. Experts in behavioural psychology, physical activity, exercise and mental health, exercise physiology, cognitive ageing, co-design, community engagement, nutrition, and exercise science were included in the research team. We acknowledge that these disciplinary backgrounds may have oriented the analysis toward particular framings, such as behaviour change, participatory processes, and implementation feasibility. The steps taken to mitigate this are described in the analysis approach section.

The University of South Australia Human Research Ethics Committee approved this study (Ethics Protocol 206129). All participants provided written informed consent before engaging in workshop activities, which included the option to be acknowledged in any resulting publications. Those who consented to acknowledgement are listed in the Acknowledgements section. No compensation was provided for participation in this workshop session. Written responses were anonymised. Ethical guidelines for responsible research were followed throughout the study (15). This manuscript has been prepared in line with the consolidated criteria for reporting qualitative research (COREQ) guidelines (16).

### Overview of the workshop and participants

The study was conducted during a single structured workshop (90 min duration) at the South Australian Rural Health Research and Education Conference in Renmark, South Australia on August 24th, 2024, hosted by the Riverland Mallee Coorong Local Health Network's Riverland Academy of Clinical Excellence. The workshop session was held mid-morning on the first day and sought to explore participants’ attitudes to technology generally and AI specifically in healthcare services, with a particular focus on the provision of regional and remote healthcare. Participants were recruited through an open call to conference attendees, who comprised healthcare professionals, researchers, and students with direct interest and experience in rural healthcare delivery. Conference attendees who did not wish to be part of the study were still able to engage in the activities but were instructed to withhold their contributions from the documentation process.

The workshop was co-facilitated by two members of the research team. Associate Professor in Design and Health Aaron Davis (AD) PhD specialises in co-design, participatory futures, and design for health. He developed the workshop materials and acted as session moderator. Tara Cain (TC) is a research project manager with expertise in nutrition, exercise science, and workshop facilitation. She coordinated participant recruitment and co-facilitated the session. No prior relationships existed between facilitators and participants beyond professional conference engagement.

Four sequential activities were planned: historical events mapping, future event possibilities, experiential futures scenarios and priority setting (Table 1). Templates used for each activity are provided as Supplementary Materials to support reproducibility and assist researchers designing similar participatory foresight workshops. Participants were seated around tables in groups of 8–10 people. AD and TC sought to actively engage with participants and assist their idea generation, helping participants move from an explicit framing of knowledge to the expression of tacit, latent and cultural knowledges through the card-based activities (15).

**Table 1:** Workshop structure.

Item	Activity	Time (minutes)	Description	Output
1	Historical events mapping	15	Participants individually documented significant events or changes in rural healthcare over the past 20 years on impactful event cards and placed them on a shared timeline.	Individual handwritten cards; collaborative timeline spanning past 20 + years.
2	Future event possibilities	25	Participants generated AI and healthcare-related future headlines using structured event cards designed to prompt divergent thinking and scenario creation.	Individual handwritten event cards describing speculative future headlines.
3	Experiential future scenarios	20	Small groups developed detailed experiential scenarios based on selected headlines, using a laddered experiential future scenarios template to explore future contexts.	Individual handwritten experiential futures templates exploring future healthcare scenarios.
4	Priority setting	15	Small groups categorised priorities for AI integration in healthcare using the MoSCoW framework.	Group-prioritised MoSCoW matrices outlining must/should/could/won't-have features of AI.

### Data collection

We designed data collection to foreground participant perspectives, using participant-led documentation as the primary recording method. Stakeholders were provided with cards and templates for all activities to prompt and collect handwritten responses. AD provided context for each activity, ensuring participants understood the frameworks that were being used, and subsequently provided further explanation and instruction as required to individuals and small groups. Photographs were taken of the timeline mapping activity. No audio or video recordings were made to ensure participant comfort, open discussion, and to privilege the participant-led documentation. No further documentation was gathered beyond workshop activities. Consent forms collected institutional affiliation, email, and signature. Individual demographic characteristics beyond institutional affiliation were not collected, consistent with the participatory and low-burden design of the workshop which prioritised open engagement and participant comfort over detailed demographic profiling.

**Figure 1:**
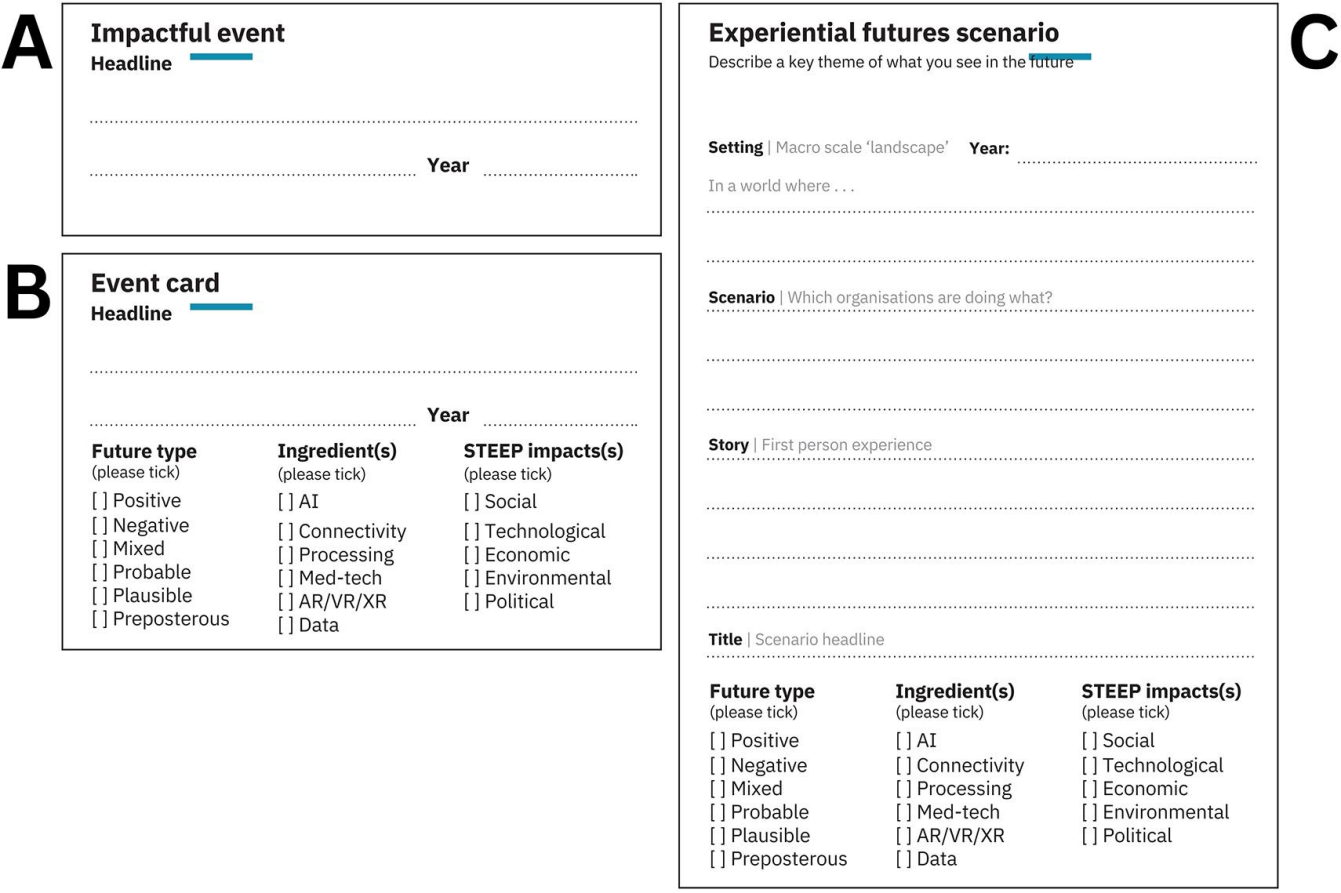
Workshop activities templates. **(A)** Historical event template to document significant historical events, technological advancements, or changes in rural health care over 20 years; **(B)** Future possibilities event card to explore potential future applications of AI and technology in rural healthcare; **(C)** Experiential futures ladder.

The workshop began with historical events mapping, where participants contributed what they saw as the most significant event, technological advancement, or change in rural healthcare over the past 20 years on a small ‘impactful event’ card. Participants were then asked to place these events on a large-scale timeline that was set up to one side of the room. The timeline included time points covering the past 20 years, followed by an illustration of a futures cone (14) stretching out approximately 80 years. This historical event activity acted as both an introduction to the event-card based timeline mapping activity, and as an icebreaker with a low entry threshold to introduce participants to others at their tables. The second activity asked participants to explore future possibilities of AI and technology in healthcare. This activity used the previously introduced format of the event-card, with added detail to prompt reflection and thinking, and to encourage more divergent idea generation. Participants were encouraged to complete as many event cards as possible, and to develop ideas that responded to different combinations of prompts from the cards. After a brief sharing discussion, and a 15-minute break, participants engaged in a third activity. For this activity, participants formed small groups of 2–3 people. These small groups reviewed cards that had been generated by the wider group and translated (and further developed) some of these into a series of experiential futures scenarios. The template provided used an experiential futures ladder structure (17) to help participants to explore the hypothetical context within which the specific event card(s) may be occurring, and to develop more tangible descriptions of possible futures. Card templates used for activities one, two, and three are shown in Figure 1. The final activity was a priority setting task using the MoSCoW framework of ‘must have’, ‘should have’, ‘could have’, ‘won't have’ (18). During this task participants remained in small groups to identify their priorities as they related to AI integration in healthcare and were directed to bias toward higher priority ranking, i.e., even if only one person on the table ranked something as ‘must have’, it was included as ‘must have’.

### Analysis approach

After the workshop, all written materials, cards and templates were collected, scanned and transcribed verbatim into a password-protected Microsoft Excel (version 2408) workbook stored on the university's encrypted cloud storage system with access limited to the research team. Two researchers independently verified transcriptions against original cards to ensure accuracy.

Data were analysed using reflexive thematic analysis (19), underpinned by a constructivist epistemology that acknowledges multiple, contextually situated realities. In this approach, quality is ensured not through claims of objectivity but through transparency, researcher reflexivity, and analytical rigour. We acknowledge that themes are constructed through the researchers' interpretive engagement with the data, not discovered as pre-existing patterns within it. We first applied an inductive analytic approach to the MoSCoW priority-setting data, allowing patterns and meanings to emerge from the data without reliance on a predefined coding structure. Consistent with reflexive thematic analysis, we did not seek data saturation; instead, adequacy was determined by the richness, diversity, and coherence of insights across workshop activities. This involved familiarisation with the data, generating initial codes, constructing candidate themes, and iteratively reviewing and refining them. Six researchers independently coded the MoSCoW responses before meeting to compare and discuss their individual codes, identifying areas of convergence and divergence. Through collaborative discussion, related codes were grouped into broader patterns, which were then developed into candidate themes. These candidate themes were iteratively reviewed and refined against the data until the team reached consensus on a coherent thematic framework. Once established, this framework was applied deductively to the remaining workshop outputs (historical event mapping, possible futures headlines, and experiential future scenarios) to explore resonance, divergence, and thematic consistency across the dataset. The themes developed were evident across all workshop activities, although their emphasis varied by task. Thematic importance was established through iterative team discussion, considering patterned meaning and relevance to the research question rather than numerical frequency alone.

An audit trail was maintained documenting analytical decisions and thematic development. Researcher reflexivity was practiced through regular team debriefings where potential biases in interpretation were critically examined.

## Findings

### Participant characteristics

Forty stakeholders participated, including academic researchers, healthcare administrators, general practitioners, clinicians, and medical and health sciences students. Participants represented universities (University of South Australia, Flinders University, University of Adelaide, University of Oxford, Columbia University), local health networks (LHNs) (Riverland Mallee Coorong LHN, Barossa Hills Fleurieu LHN), government departments (SA Health, Department for Health and Wellbeing), and a research institute (South Australian Health and Medical Research Institute).

### Thematic analysis

**Figure 2:**
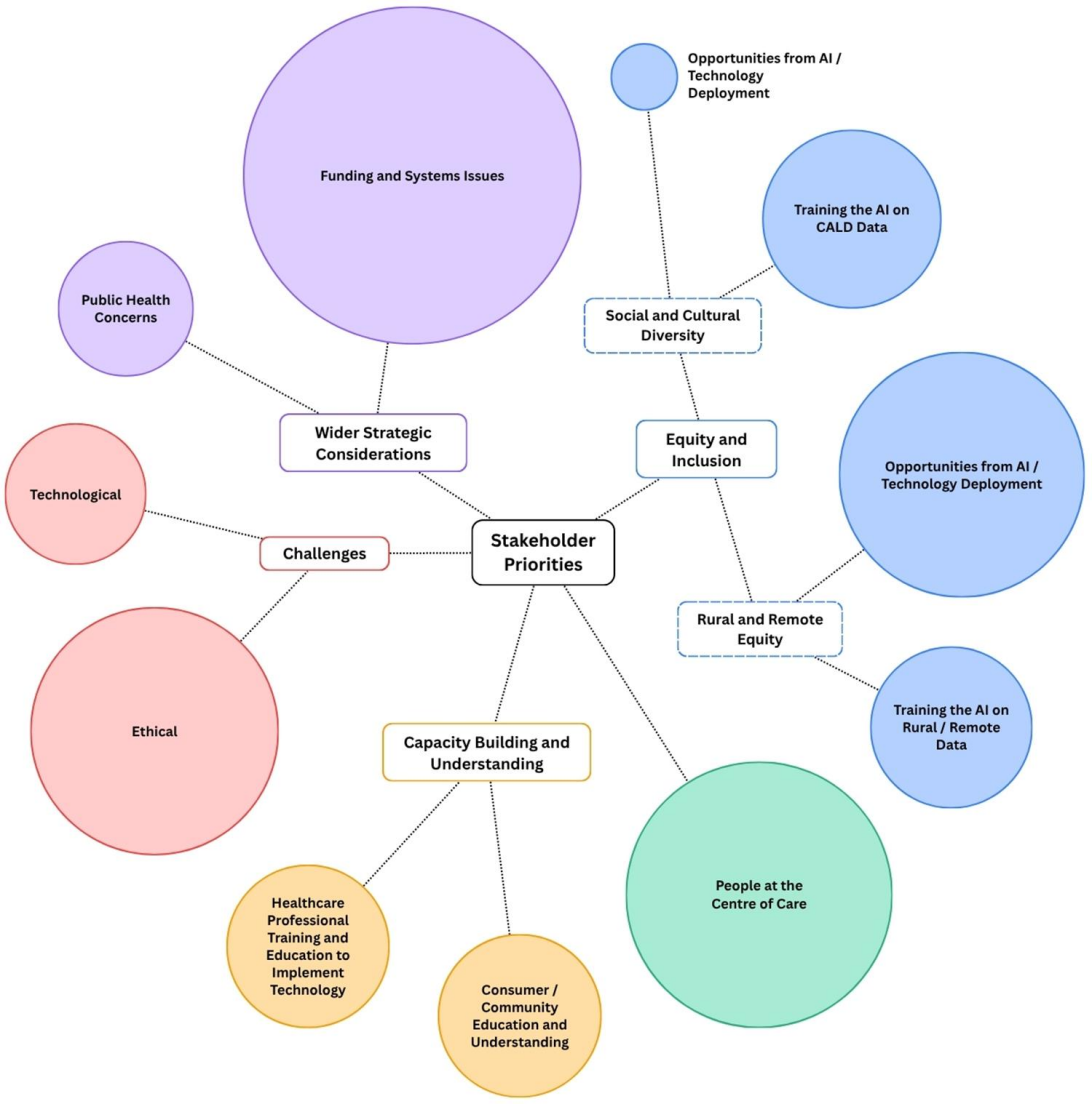
Thematic map of stakeholder priorities for AI integration in rural healthcare. The visual representation of the thematic analysis highlights four prominent themes: opportunities from AI and technology deployment viewed through a rural equity lens, people at the centre of care, ethical challenges, and funding and systems issues arising from wider strategic considerations. The size of each circle reflects the relative frequency of data excerpts coded to each theme within the MoSCoW priority setting activity.

We collected a total of 47 historical event cards, 192 future event cards, 15 experiential futures scenarios, and eight MoSCoW activity sheets. From this dataset, we identified five primary themes describing stakeholder priorities for the future of AI in rural healthcare: equity and inclusion, people at the centre of care, capacity building and understanding, challenges, and wider strategic considerations. Subthemes associated with each theme are shown in Figure 2. The size of each circle reflects the relative frequency of excerpts coded to that theme within the MoSCoW activity; these visuals are intended to show qualitative emphasis rather than quantitative weight, so numerical counts are not displayed. A supplementary table reporting the number of data excerpts coded to each theme and subtheme within the MoSCoW priority-setting activity is provided in Supplementary File 2 for transparency.

Across the full dataset, four themes stood out: i) equity and inclusion (with a particular focus on rural and remote equity and the opportunities created by AI and technology deployment, ii) people at the centre of care, iii) ethical challenges, and iv) funding and systems issues within the wider strategic context. Together, these themes capture the key priorities and guardrails that participants viewed as essential for the future integration of AI in rural healthcare. All excerpts presented in the findings are verbatim. Notably, participants used ‘AI’ broadly throughout the workshop activities, encompassing a range of technologies including clinical decision support, remote monitoring and diagnostics, predictive analytics, digital twins, and automated administrative functions. This reflects how AI is commonly discussed in both public and professional discourse and should be considered when interpreting the findings.

### Theme 1: opportunities from AI and technology deployment through a rural equity lens

#### Must haves

Participants identified several must haves for AI and technology deployment in rural healthcare with a strong focus on reducing geographic disadvantage. Priorities included “*reducing the need for travel to receive services by increasing telehealth/AI”, “how do we improve imaging to reduce the need to travel”, “access to healthcare”, “reducing waiting times”, “equity of access for rural people”, “people who cannot access healthcare”, “technology intervention”,* and *“affordable health care for those that can least afford it”.* These insights reflect a clear alignment between AI opportunities and the most persistent barriers in rural healthcare: geographic distance, workforce shortages, and the cost of accessing care.

These priorities drew on past technological progress in rural health. Participants pointed to “*digital technology advancements…* [that] *have enabled a range of services to now be more accessible”,* “*integrating telehealth for real time specialist review in emergency presentations i.e., code stroke, cardiac arrest improving health outcomes/survivorship post event”,* and “*implementation of SAVES in rural emergency departments”* (the South Australian Virtual Emergency Service). At the same time, they noted that significant limitations remain, particularly the inequities highlighted during COVID-19: “*This has changed the way we view healthcare accessibility, the underlying inequities between rural and remote regions and metro areas”.*

Looking forward, participants’ future headlines imagined technological advances that directly addressed these barriers, particularly geographic distance and limited diagnostic capacity, such as “*first surgery performed purely remotely”, “whole-body health scanner diagnoses any health condition in 1 min”,* and “*teleportation eliminates transportation barrier for rural residents to access specialist medical care”.* In the experiential future scenario activity, participants extended these opportunities beyond access, envisioning AI supporting prevention and personalised care in contexts where workforce constraints limit ongoing chronic disease management: “*using AI advantageously for prevention of chronic conditions”, “when experimenting a new drug in pt w* [patients with] *diabetes, provide drug to digital twin to assess side effects…”,* and “*rural Australian locations technology makes a positive contribution to health and wellbeing; healthcare organisations are using technology to prevent ill-health and cure diseases”.*

However, participants also imagined futures where these opportunities fail to materialise because broader system issues remain unresolved. Cautionary scenarios described worsening access, with one group depicting: “*a bleak future*: *waiting 20 h to get into hospital and 1 year to see a GP* [General Practitioner]*. Patients now treat themselves via AI self-management tools and get medicine on the black market”.*

### Theme 2: people at the centre of care

#### Must haves

Participants strongly emphasised that AI must support, not replace, the human elements of healthcare. Must haves included “*retain first-person care in rural communities”, “tools development must be person-centric approaches”, “person centred care - treat the person not the disease”,* and “*use AI to augment rather than replace healthcare”.* They also highlighted the importance of “*individual control of AI”*, the need to ensure that “*the enhancement of technology does not replace an increase in health professionals in rural areas”,* and the continued value of “*local knowledge and skills”* and “*workforce - local people in health systems”*.

Historical reflections provided some grounding for these views, including the growing emphasis on “*evidence based practice…* [and] *personalised medicine”.* Participants’ future focused ideas often described AI enhancing rather than diminishing human care. Examples included “*no! to nursing homes: med-tech and machine learning allowing older adults to safely stay at home”,* “*rural populations offered VR telehealth consults with real healthcare providers”,* and *“AI to slash administrative burden: health workers find more time for F2F* [face to face] *care as AI records, synthesises & types their notes for them”.* Experiential scenarios also explored personalised and holistic possibilities, such as *“implantable sensor for precision nutrition - scan each day to see what your needs are”,* and broader system reform: *“Healthcare has become number 1 priority in the funding budget. LCLHN* [Limestone Coast Local Health Network] *offer permanent & ongoing contracts & have 1:2 nursing patient ratio. Patients are able to receive individualised & personalised care for the entirety of their stay”.*

Participants also described futures where patients have greater autonomy over their information and care. Future headlines included “*open disclosure of medical records to the public - can't remember what your Dr told you? Don't worry from now on every clinic note is in your hands”* and *“universal electronic health records improve patient care”.* Others imagined integrated, holistic, and community-centred healthcare models: “*integrated holistic healthcare services developed in rural/remote regions”, “health services to focus on health not illness”, “health is reconceived within a community paradigm”,* and *“lived experience based co-designed clinical guidelines not only clinician data driven”.*

However, participants also imagined consequences when person-centred care is eroded. One scenario portrayed an older adult unable to access human care despite having comprehensive home monitoring, concluding bleakly: “*Call the undertaker not the ambulance”.* Another explored the loss of human connection in technologically saturated futures, such as “*complete IT connectivity and increased individual isolation”*, an emerging “*gap between tech-enabled and tech-disenfranchised”*, and people described as “*disempowered ‘healthy’ (biologically) but disconnected from the world ‘unhealthy’ (psychosocially)”*. A similarly stark scenario described “*healthy man found in coma, 37 days since last human contact”*.

#### Won't haves

Participants were clear about boundaries. AI must not lead to “*eliminating human context”* or “*eliminating human doctors”.* They also expressed concerns about social trade-offs, such as “*trade-offs between social norms and individualised liberties”*, and caution about futures where technological over-reliance diminishes core human elements. One headline captured public resistance: “*swinging back. Super sub specialists boycotted by patients wanting to see real Drs”*.

### Theme 3: ethical challenges

#### Must haves

Participants identified strong ethical guardrails for the future development of AI and digital technologies in healthcare. Must haves included addressing “*privacy breach”, “confidentiality around the individual & community data lakes* [leaks]*”, “misinformation spread by AI”,* “*better regulation of AI as it becomes more advanced”,* and *“appropriate rural & indigenous ethical frameworks & processes for healthcare”.* Participants also highlighted environmental considerations, noting the need for “*reduced waste prioritising reusable energy sources”* and “*reusable & sustainable energy sources of medical equipment and technology”.*

Future headlines reflected concerns about what might occur if these issues are not properly addressed. Participants imagined scenarios such as “*hackers infiltrate Australia's health records database and launch targeted biochemical attack”, “AI data base breached”,* and *“life insurance companies know your disease before you do”.* At the same time, some headlines presented more hopeful possibilities, including approaches that promote transparency and autonomy: “*My Data not My Gov: individual ownership of all personal health related data that I choose to share across the spectrum of clinicians and researchers”.*

Participants also raised concerns about system reliance and the possibility of governance failures. Examples included “*big IT crash brings health system into chaos: clinicians unable to make decisions without AI”, “humans can no longer control artificial intelligence”,* and *“gene testing misdiagnoses community due to polymorphic genes”.* Participants imagined breakdowns in ethical oversight: “*HREC* [Human Research Ethics Committee] *found to be using AI to shortcut approvals”,* and failures in duty of care: “*deaths in custody increase due to Health Services washing their hands of ‘behaviour problems' and clearing unwell patients for custody”.*

#### Won't haves

Participants were clear about ethical boundaries they felt must not be crossed. These included: “*involuntary euthanasia of our elderly population due to costs of aged care”, “genetic selection in children – antenatal”,* and “*one child policy in Australia to address population growth”.* These concerns pointed to broader fears about autonomy, coercion, and the potential misuse of powerful technologies.

Future event possibilities highlighted the risks associated with losing control over personal freedoms, such as “*AI dictates daily behaviour on me/you to maximise current & future health”, “human emotions outdated by AI.* *..human connections/feelings/experiences no longer relevant”,* and *“full integration of tech AI and humans. From birth, implants are put linking all humans to network”.*

Experiential futures scenarios illustrated more extreme versions of these ethical concerns. In one scenario participants described a future with little personal agency: “*no need to access specialists, behaviour is determined by ‘best healthcare’ actions: minimising liberties. AI overlords”.* Another imagined complete loss of human control and agency: “*AI has taken over healthcare. No more Drs or rural medical schools. AI is diagnosing, treating, prescribing, manipulating. Increased suicide rates due to social isolation. AI is controlling the human population with medication, propaganda, food etc”.*

### Theme 4: funding and systems issues

#### Must haves

Participants identified a range of system-level requirements that would need to accompany AI and technology integration in rural healthcare. Funding was a central concern, with calls for *“increased funding for health services”, “increased funding for healthcare positions after uni* [university]*”, “equitable funding for rural health”,* and “*fund caring in families”.* Participants also highlighted the need for investment in technology-enabled care at home, including “*increase funding for at home self monitoring of health conditions, BP/vital signs”.*

Beyond funding, participants emphasised broader system capacity. Must haves included “*better aged care facilities”, “increase GP's in all of Australia”, “establish a research unit in each LHN”,* and “*increase clinical trial activity in each LHN”.* Infrastructure equity was also a priority, reflected in comments such as “*equitable access to diagnostic tools”, “inequitable access to health technology i.e., all hospitals require CT scan/radiology”,* and the need for fair “*geographical distribution of infrastructure, resources and opportunities”.* Workforce and governance considerations were also identified, including the importance of a strong “*rural workforce in all professions”,* ensuring “*political agendas address rural issues”,* and improving “*communication across sectors”.*

These concerns were grounded in historical reflections that highlighted long-standing structural issues. Participants recalled tensions around resource allocation and the organisation of care, noting examples such as “*helipad to impact health budget, but saves lives”, “increased demand of/on health services/GP's”, “the legal pressure forcing doctors to specialise… too costly to be a generalist”,* and “*reduced GP involvement in hospital care ∼10–15 years”.*

Participants’ future headlines imagined more equitable and better resourced health systems. Examples included “*uncapped budget/funding for healthcare”, “health adequately funded to provide culturally appropriate care for all patients”, “increased funding for mental health & CAMHS* [Children and Adolescent Mental Health Service] *in South Australia”,* and *“research is funded on a model equivalent to teaching”.* Research and innovation capacity also featured, with future possibilities such as “*all South Australian regional communities have access to clinical trials through successful implementation of teletrials. No one shall miss out on advanced treatment”,* and “*rural populations reach parity in clinical trials to metro counterparts”.*

#### Won't haves

Participants also identified system configurations they believed must be avoided, particularly those that further marginalise rural communities. These included “*everything happens in the city only”* and “*if politicians fail to recognise rural needs & decisions are not rural inclusive”.* Such concerns were echoed in historical reflections describing persistent inequities, such as “*access to services* [has] *forever been* [a] *major issue for rural & remote communities”,* and “*global pandemic isolates isolated communities even more”.*

Future event possibilities illustrated the consequences of failing to invest adequately in rural health systems. Examples included “*last rural hospital closes in South Australia creating metropolitan hospital super hubs”,* and *“‘rural medicine now extinct’ - all patients teleport to central metro service”.*

## Discussion

This futures-focused participatory workshop revealed that stakeholders view AI as offering substantial potential to improve rural healthcare, particularly by reducing travel, increasing access to specialist input, and strengthening preventive and personalised care. At the same time, participants stressed that these benefits are contingent on addressing long-standing structural barriers, including digital connectivity, affordability, workforce shortages, and uneven distribution of diagnostic and digital infrastructure. Equity concerns operated across both individual access to services and community-level capacity, with participants emphasising that reducing barriers for individuals must be accompanied by systemic investment in the infrastructure, workforce, and funding models that sustain rural health systems. Across all activities, participants prioritised person-centred care, emphasising that AI should augment rather than replace human clinicians and must preserve trust, relationships, and local knowledge. Ethical considerations were central, with concerns focused on data privacy, governance, cultural safety, transparency, and the risks of over-reliance on automated systems. Finally, participants highlighted that technology alone cannot address rural health inequities; meaningful progress requires sustained systemic investment, participatory governance, and policy frameworks that centre rural communities in the design and deployment of AI.

Participants identified geographic inequity as a defining barrier to healthcare access and viewed AI as a potential mechanism to help bridge this divide, particularly through reducing travel, improving diagnostic capacity, and expanding virtual service delivery. Broader evidence shows that AI-enabled innovations such as remote monitoring and telemedicine can enhance access by connecting rural patients with specialist services without the burden of travel (20). Emerging Australian examples illustrate this potential: in remote Western Australia, a co-designed mobile AI-assisted diabetic retinopathy screening model achieved an 11-fold increase in screening rates compared to existing primary care-based models, with 96% of patients reporting satisfaction with the use of AI (21). Similarly, Habib et al. (2022) developed a computer-vision algorithm trained on over 6,500 otoscopic images from Aboriginal and Torres Strait Islander children in rural and remote Northern Territory, achieving up to 99.3% accuracy in classifying ear disease, a tool designed to support frontline health workers in triaging ear conditions without requiring specialist access (22). Yet, these benefits depend on structural readiness. The rapid transition to telehealth during the COVID-19 pandemic demonstrated both the promise and fragility of digital access. While telehealth improved continuity of care, it also widened inequities among those without stable internet, adequate devices, or digital literacy (23). In Australia, these barriers disproportionately affect rural, low-income, and Aboriginal and Torres Strait Islander communities (24). Global evidence confirms that infrastructure deficits, affordability, and limited technical capacity remain persistent obstacles to equitable AI adoption (25). Participants’ emphasis on affordability and local capability echoes calls for investment beyond technology procurement to include broadband access, workforce training, and cost-effective deployment (25). Without these foundations, even advanced AI systems risk reinforcing existing disparities. Policymakers must therefore prioritise infrastructure and digital literacy as prerequisites for AI deployment, linking adoption to parallel investment in connectivity, devices, and community education.

Participants emphasised that while AI may enhance efficiency, it must not erode the human relationships that underpin rural healthcare. Calls to “*retain first-person care*,” “*treat the person, not the disease*,” and “*use AI to augment rather than replace healthcare*” reflected a shared belief that trust, empathy, and continuity remain central to quality care. This perspective aligns with evidence showing that successful digital health integration depends on preserving relational dimensions of practice. Lupton's (2017) concept of ‘affective atmospheres', describes how digital technologies shape the emotional and sensory dimensions of care encounters, arguing that AI's success depends not only on technical accuracy but also on sustaining feelings of safety and connection that characterise compassionate practice (26). Empirical studies similarly show that patients find remote interventions meaningful when they preserve personal commitment and partnership with trusted professionals, with telephone-based contact to date fostering greater perceptions of safety and engagement than automated functions (27). The use of co-design and participatory approaches in designing future health systems are critical to maintaining these relational foundations (28). Future scenarios describing isolation and disconnection reflected participants’ discomfort with depersonalised models of care. This concern is particularly salient given rural workforce shortages (29), which may create pressure to use AI as a substitute rather than a support for clinicians, potentially undermining the trusted relationships participants valued.

Participants identified ethical considerations; privacy, consent, and transparency, as central to whether AI would be trusted within rural healthcare. Concerns about confidentiality, governance failures, and data misuse reflected a desire for safeguards that protect individuals while promoting collective benefit. Trust in digital health systems depends on transparent governance, equitable oversight, and sustained public accountability (10). Technocentric approaches that ignore social and moral contexts create “blind spots” limiting responsiveness to community values (10), and AI without strong ethical frameworks risks eroding professional accountability and amplifying inequities (30). Governance must therefore be participatory, embedding mechanisms that ensure rural and marginalised voices are represented in policy and design. Cultural safety was also central to participants’ concerns. In Australia, ethical data practices must uphold Indigenous Data Sovereignty, affirming Australian First Nations peoples’ rights to control how data are collected, interpreted, and applied (31). Frameworks such as the CARE (Collective Benefit, Authority to Control, Responsibility, Ethics) and the Maiam nayri Wingara principles provide mechanisms for embedding these rights in health data governance (32). Integrating such frameworks into AI development offers a tangible pathway to culturally grounded transparency and trust, ensuring governance reflects both community values and Indigenous sovereignty.

Participants emphasised that technological innovation alone cannot resolve the structural inequities that define rural healthcare. While AI may enhance efficiency, its success depends on parallel investment in workforce, infrastructure, and funding models that address long-standing deficits in service capacity. Without systemic reform, technology risks serving as a short-term fix that leaves inequities unchanged. Persistent underinvestment continues to drive disparities in access, outcomes, and workforce distribution across rural Australia. The National Rural Health Alliance reports a funding shortfall exceeding $6.5 billion between rural and metropolitan populations, reflecting fragmented governance and inadequate investment in preventive care (33). Broader research argues that closing the rural health gap requires sustained, community-based investment and research capacity rather than reliance on top-down technological interventions (34). These systemic shortcomings may also constrain the ability of rural areas to adopt and sustain AI solutions. The Australian Medical Association likewise notes that poor digital connectivity undermines the potential of digital health tools where complementary infrastructure and workforce supports are lacking (35). Participants envisioned equitable rural healthcare requiring both innovation and targeted investment that is responsive to local needs. Approaches based on the benefits of investment in urban healthcare ‘trickling-out’ to rural populations are not fit for purpose. AI readiness in rural settings is therefore inseparable from broader systemic reform; without sustained investment in workforce, connectivity, and local service capacity, AI deployment risks widening rather than narrowing the rural-urban health divide. In many countries, rural and remote populations are recognised as priority groups within national health strategies and innovation funding schemes. Evidence from rural digital health initiatives suggests that sustained implementation is more likely when funding is policy-backed and aligned with broader system reform, rather than limited to short-term pilot grants (36, 37). Future AI initiatives in rural healthcare may therefore benefit from integration within existing rural health policy frameworks and long-term investment strategies.

These systemic challenges are compounded by a regulatory and technical landscape that remains in development. In Australia, AI-enabled clinical tools with a medical purpose are regulated as medical devices by the Therapeutic Goods Administration under a technology-agnostic framework that requires pre-market assessment and evidence of safety and performance, including transparency sufficient to enable evaluation of AI-specific risks (38). At the same time, AI models developed and validated in metropolitan settings may have limited translatability to rural health systems, where differences in clinical workflows, patient populations, and data availability can significantly affect algorithm performance (39). Participants' visions of AI-assisted diagnostics, remote monitoring, and predictive care thus intersect with real and unresolved questions about how such tools will be validated, integrated into clinical workflows, and governed in the specific contexts in which they are needed most. Policy frameworks must therefore embed AI within broader strategies for rural health equity, ensuring that digital innovation strengthens rather than substitutes for the foundational systems needed to deliver sustainable, person-centred care.

The themes identified by participants connect to several established frameworks in the AI-for-healthcare literature. Participants' consistent emphasis on human oversight and the augmentation rather than replacement of clinicians aligns with human-in-the-loop approaches, which position clinician judgement as essential in AI-assisted decision-making (40). Concerns about data governance, transparency, and accountability reflect the principles of algorithmic accountability, which call for clear mechanisms to audit, explain, and contest AI-driven decisions in health systems (41). Furthermore, participants’ focus on cultural safety, equitable access, and the risks of AI systems trained on unrepresentative data resonates with growing calls to evaluate AI beyond accuracy alone, encompassing fairness, safety, and robustness across diverse populations and settings (42). That these concerns emerged organically from rural healthcare stakeholders through a futures-oriented methodology underscores their salience and suggests that responsible AI implementation must be informed by the perspectives of those who will use and be affected by these systems.

While this study was not designed to evaluate specific technologies, the stakeholder priorities identified here suggest several areas that may warrant careful pilot testing in rural contexts. Future research might consider piloting AI applications that aim to reduce travel and diagnostic delays, such as AI-assisted triage or decision-support tools integrated into rural emergency and primary care settings. Given participants’ strong emphasis on preserving relational care, early trials could also focus on AI systems that augment clinicians rather than replace them, for example through reducing administrative burden or supporting documentation, with evaluation of impacts on clinician workload and patient experience. In addition, stakeholders highlighted the importance of transparent governance and cultural safety. Any future AI pilots in rural healthcare may benefit from embedding Indigenous Data Sovereignty principles and locally grounded governance structures from the outset.

### Strengths and limitations

This study has several strengths. The participatory foresight methodology incorporated multiple structured activities that encouraged creative and future-oriented reflection, enabling participants to move beyond conventional thinking about AI in healthcare. In particular, the futures-oriented activities surfaced concerns, such as dystopian scenarios of depersonalised care and ethical red lines around data misuse, that conventional survey or interview methods may not have elicited. Collecting data across four different activities (historical events mapping, future event possibilities, experiential future scenarios, and priority setting) allowed for triangulation of data and strengthened analytical credibility. The workshop engaged a diverse group of stakeholders with significant experience in rural healthcare, including clinicians, academics, and healthcare administrators, providing breadth of perspectives. This professional diversity enriched the findings by capturing clinical, research, and system-level viewpoints, and the themes identified reflect challenges common to rural healthcare systems internationally, suggesting potential transferability to other rural and remote contexts. Participant-led documentation enhanced trustworthiness by giving individuals control over recorded content, while independent coding and consensus discussions supported rigour.

Several limitations warrant discussion. The single-session, 90-minute format may have constrained the depth of individual reflection and limited opportunities for participants to build on one another's ideas over time. Multiple sessions would have allowed for deeper exploration and iterative development of scenarios. Conducting the workshop within a professional conference context was an intentional decision that provided access to healthcare professionals, researchers, and administrators with direct experience in rural healthcare systems. However, despite the dual role many participants played as both community members and institutional stakeholders, this approach meant the sample did not include dedicated rural health consumer voices. While the themes of person-centred care, trust, and equity reflect core professional values grounded in participants’ direct experience of delivering care in rural settings, future research should employ similar participatory foresight methods with rural health consumers to capture their lived experience of AI-mediated care. Consumer perspectives on autonomy, trust, and equity would provide essential complementary insights that extend and validate the provider and system-level priorities identified in this study. The reliance on written contributions, while fostering participant comfort and openness, limited the nuance that might have been captured through audio recordings of group discussions. Detailed individual demographic data were not collected, which limits the ability to fully characterise the sample beyond professional role and institutional affiliation. The broad use of ‘AI’ by participants, without differentiation between technology types, limits the specificity of the findings. Future research could use more targeted methods to explore stakeholder priorities in relation to distinct AI applications. Finally, while the sample included diverse professional roles, all participants had chosen to attend a rural health conference, suggesting a level of interest and engagement in rural health issues that may not be representative of all healthcare stakeholders. These factors were carefully considered when interpreting the findings.

## Conclusion

This participatory foresight workshop used futures-oriented methods to explore how rural stakeholders envision the role of AI in healthcare. Participants saw substantial potential for AI to reduce geographic barriers, improve access, and support more personalised and preventive care. They also identified important guardrails, emphasising the need to protect person-centred care, maintain strong ethical and governance frameworks, and address long-standing system challenges in funding, workforce, and digital and diagnostic infrastructure. Overall, the findings suggest that AI can contribute to better health outcomes for rural communities, but only when implementation is grounded in local priorities and supported by broader system investment. Together, the themes and priorities identified in this study offer a stakeholder-informed foundation for developing context-sensitive implementation pathways for AI in rural healthcare. Futures-based, co-design approaches such as this can help guide responsible integration of emerging technologies in rural healthcare settings.

## Data Availability

The raw data supporting the conclusions of this article will be made available by the authors, without undue reservation.
