# Supplementary material for: Exploring plausible futures for artificial intelligence in rural healthcare: insights from participatory foresight methods
Source: Front Digit Health. 2026 Apr 13;8:1750172. doi: 10.3389/fdgth.2026.1750172 (PMC13111446; doi:10.3389/fdgth.2026.1750172)
Supplement: Supplementary file 2 [file Datasheet1.pdf]

## Impactful event

**Headline**

..........................................................................................

.................................................................. **Year** ...............................


## Impactful event

**Headline**

..........................................................................................

.................................................................. **Year** ...............................

## Impactful event

**Headline**

..........................................................................................

.................................................................. **Year** ...............................

## Impactful event

**Headline**

..........................................................................................

.................................................................. **Year** ...............................

## Impactful event

**Headline**

..........................................................................................

.................................................................. **Year** ...............................

## Impactful event

**Headline**

..........................................................................................

.................................................................. **Year** ...............................

## Event card

**Headline**

**Year**

**Future type**
(please tick)

[ ] Positive
[ ] Negative
[ ] Mixed
[ ] Probable
[ ] Plausible
[ ] Preposterous

**Ingredient(s)**
(please tick)

[ ] AI
[ ] Connectivity
[ ] Processing
[ ] Med-tech
[ ] AR/VR/XR
[ ] Data

**STEEP impacts(s)**
(please tick)

[ ] Social
[ ] Technological
[ ] Economic
[ ] Environmental
[ ] Political


## Event card

**Headline**

**Year**

**Future type**
(please tick)

[ ] Positive
[ ] Negative
[ ] Mixed
[ ] Probable
[ ] Plausible
[ ] Preposterous

**Ingredient(s)**
(please tick)

[ ] AI
[ ] Connectivity
[ ] Processing
[ ] Med-tech
[ ] AR/VR/XR
[ ] Data

**STEEP impacts(s)**
(please tick)

[ ] Social
[ ] Technological
[ ] Economic
[ ] Environmental
[ ] Political

## Event card

**Headline**

**Year**

**Future type**
(please tick)

[ ] Positive
[ ] Negative
[ ] Mixed
[ ] Probable
[ ] Plausible
[ ] Preposterous

**Ingredient(s)**
(please tick)

[ ] AI
[ ] Connectivity
[ ] Processing
[ ] Med-tech
[ ] AR/VR/XR
[ ] Data

**STEEP impacts(s)**
(please tick)

[ ] Social
[ ] Technological
[ ] Economic
[ ] Environmental
[ ] Political

## Event card

**Headline**

**Year**

**Future type**
(please tick)

[ ] Positive
[ ] Negative
[ ] Mixed
[ ] Probable
[ ] Plausible
[ ] Preposterous

**Ingredient(s)**
(please tick)

[ ] AI
[ ] Connectivity
[ ] Processing
[ ] Med-tech
[ ] AR/VR/XR
[ ] Data

**STEEP impacts(s)**
(please tick)

[ ] Social
[ ] Technological
[ ] Economic
[ ] Environmental
[ ] Political

## Experiential futures scenario

Describe a key theme of what you see in the future

**Setting** | Macro scale 'landscape' **Year:** ........................................

In a world where . . .

........................................................................................

........................................................................................

**Scenario** | Which organisations are doing what?

........................................................................................

........................................................................................

**Story** | First person experience

........................................................................................

........................................................................................

........................................................................................

**Title** | Scenario headline

........................................................................................

**Future type** (please tick)

[ ] Positive
[ ] Negative
[ ] Mixed
[ ] Probable
[ ] Plausible
[ ] Preposterous

**Ingredient(s)** (please tick)

[ ] AI
[ ] Connectivity
[ ] Processing
[ ] Med-tech
[ ] AR/VR/XR
[ ] Data

**STEEP impacts(s)** (please tick)

[ ] Social
[ ] Technological
[ ] Economic
[ ] Environmental
[ ] Political


## Experiential futures scenario

Describe a key theme of what you see in the future

**Setting** | Macro scale 'landscape' **Year:** ........................................

In a world where . . .

........................................................................................

........................................................................................

**Scenario** | Which organisations are doing what?

........................................................................................

........................................................................................

**Story** | First person experience

........................................................................................

........................................................................................

........................................................................................

**Title** | Scenario headline

........................................................................................

**Future type** (please tick)

[ ] Positive
[ ] Negative
[ ] Mixed
[ ] Probable
[ ] Plausible
[ ] Preposterous

**Ingredient(s)** (please tick)

[ ] AI
[ ] Connectivity
[ ] Processing
[ ] Med-tech
[ ] AR/VR/XR
[ ] Data

**STEEP impacts(s)** (please tick)

[ ] Social
[ ] Technological
[ ] Economic
[ ] Environmental
[ ] Political

| Must Address | Should Address | Could Address | Won’t Address |
|---|---|---|---|
| | | | |

## Reflection

**Objective** | What did you see and hear?

**Reflective** | What did you feel?

**Interpretive** | What was your key insight?

**Decisional** | What should be taken forward?


## Reflection

**Objective** | What did you see and hear?

**Reflective** | What did you feel?

**Interpretive** | What was your key insight?

**Decisional** | What should be taken forward?

## Reflection

**Objective** | What did you see and hear?

**Reflective** | What did you feel?

**Interpretive** | What was your key insight?

**Decisional** | What should be taken forward?

## Reflection

**Objective** | What did you see and hear?

**Reflective** | What did you feel?

**Interpretive** | What was your key insight?

**Decisional** | What should be taken forward?
